# Uber use after alcohol consumption among car/motorcycle drivers in ten Brazilian capitals

**DOI:** 10.11606/s1518-8787.2023057005147

**Published:** 2023-10-30

**Authors:** Érika Carvalho de Aquino, Otaliba Libânio de Morais

**Affiliations:** I Universidade Federal de Goiás Instituto de Patologia Tropical e Saúde Pública Departamento de Epidemiologia Goiânia GO Brasil Universidade Federal de Goiás . Instituto de Patologia Tropical e Saúde Pública . Departamento de Epidemiologia . Goiânia , GO , Brasil

**Keywords:** Alcohol Drinking, Driving Under the Influence

## Abstract

**OBJECTIVE:**

This study aimed to measure the proportion of Uber use instead of drinking and driving in ten Brazilian capitals, in 2019.

**METHODS:**

A cross-sectional survey was developed in ten Brazilian capitals. Data were collected in agglomeration points (AP) and sobriety checkpoints (SC). Based on responses to a standardized questionnaire, the proportion of drivers who used Uber instead of drinking and driving was measured for total sample of each methodology and stratified by municipality, age group, gender, education level, and type of vehicle. Fisher’s exact test was used to make comparisons between the strata.

**RESULTS:**

A total of 8,864 drivers were interviewed. The most used means of transport to replace driving after drinking alcohol was the Uber system (AP: 54.6%; 95%CI: 51.2–58.0. SC: 58.6%; 95%CI: 55.2–61.9). Most of these users were aged from 18 to 29 years, women, with at least one higher education degree. According to the AP methodology, the highest magnitude of this indicator was found in Vitória (ES) (71.0%; 95%CI: 63.5–77.5), whereas the lowest was observed in Teresina (PI) (33.1%; 95%CI: 22.7–45.5). According to the SC methodology, the highest magnitude of the indicator was also found in Vitória (ES) (78.3%; 95%CI: 68.8–85.5), whereas the lowest was observed in Boa Vista (RR) (36.6%; 95%CI: 26.8–47.7).

**CONCLUSION:**

In Brazilian capitals, the study showed higher proportions of Uber use instead of drinking and driving. This type of scientific evidence on factors associated with road traffic injuries presents the potential to guide public health interventions.

## INTRODUCTION

Every year, about 1.35 million deaths are reported from road traffic injuries (RTI) worldwide, being the main cause of death among people aged from 5 to 29 years. In addition to high mortality, RTI are responsible for 20 to 50 million non-fatal injuries annually, many of them resulting in permanent disability ^1^ . Therefore, the risk factors and scenarios associated with RTI need to be studied and understood for the planning of public policies designed to mitigate them ^2^ .

Drinking and driving is one of the main risk factors for RTI worldwide. It is estimated that 20% of drivers involved in fatal accidents have some level of alcohol content during the event ^3^ . In some low and middle-income countries, these numbers can reach 69% ^3^ . In 2016, more than 170,000 deaths were recorded from land transport accidents related to alcohol consumption. Apart from high morbidity and social risks, it is estimated that 3.9 deaths can be attributable to alcohol consumption per 100,000 inhabitants annually in Brazil ^4^ . Alcohol use is an important risk factor for external causes of death, especially those resulting from RTI ^5^ .

Moreover, in Brazil, to inhibit the consumption of alcoholic beverages by motor vehicle drivers, Law nº 11,705 ( *Lei Seca* ) was enacted in 2008, establishing penalties for this type of infraction ^7^ . However, the impact of this law lasted only a few months due to the lack of inspection equipment to measure the blood alcohol concentration (BAC) in drivers ^8^ . The new *Lei Seca* nº 12.760, 2012 ^9^ (Prohibition Law) was enacted in a new scenario, in which the inspection of drivers was possible and stricter penalties were imposed on drivers with positive BAC. Shults et al. ^10^ stated that this type of measure can reduce the number of traffic accidents up to 24%.

In 2010, the Uber system was launched, a digital platform for smartphone and tablet devices ^11 , 12^ that supports a type of private transport service such as e-hailing or ridesourcing ^11 , 13^ . In Brazil, Uber started its activities in 2014 ^13^ . Then, in the same year, it began operating in São Paulo, Belo Horizonte, and Brasília ^14^ . Currently, the app is available in more than 500 Brazilian municipalities, with 1 million drivers and 30 million registered users ^15^ .

The rapid expansion of Uber Technologies Incorporated and the e-hailing application transformed the logistics network and urban mobility ^16^ . Although the final objective of this system is not focused on traffic safety, studies have shown that it can affect its intermediate indicators, such as the prevalence of driving and drinking. In this case, there could be repercussions on the final traffic safety indicators, such as mortality and hospitalization rates due to RTI ^17 , 18^ . Some studies show an association between the implementation of the Uber system and the reduction in the prevalence of driving after drinking alcohol, which is one of the main risk factors for morbidity and mortality from RTI ^18^ . However, other studies have not detected this impact, or even an increase in the magnitude of this indicator after the implementation of the Uber system ^17 , 23 , 24^ . In Brazil, only one study was published on the impact of the Uber System on morbidity and mortality from traffic injuries ^25^ , and there are no studies that address this impact on the prevalence of driving after drinking alcohol.

The introduction of new interventions related to traffic must be systematically monitored and evaluated ^26^ . Thus, due to contradictory results and the scarcity of studies on the impact of the Uber system implementation on the prevalence of driving after drinking alcohol in Brazil, this study aimed at measuring the proportion of Uber use among drivers of cars and motorcycles after drinking alcohol in ten Brazilian capitals in 2019.

## METHODS

### Study Design and Locations

A cross-sectional survey was conducted on the sociodemographic characteristics and background of car and motorcycle drivers, as well as possible risk factors for RTI, in ten Brazilian capitals, in 2019. The study was developed in the following Brazilian capitals: Belo Horizonte, Boa Vista, Cuiabá, Florianópolis, Macapá, Salvador, São Luís, São Paulo, Teresina, and Vitória. These municipalities represent the five Brazilian regions and currently account for more than 22 million inhabitants.

### Target Population, Inclusion, and Exclusion Criteria

Participants were included based on the following criteria: being 18 years or older, driving a car or motorcycle, and living in one of the investigated municipalities. Commercial vehicle drivers (taxi, mototaxi, bus, application vehicles, heavy vehicles, and delivery vehicles) were not eligible for survey data collection.

**Figure 1:**
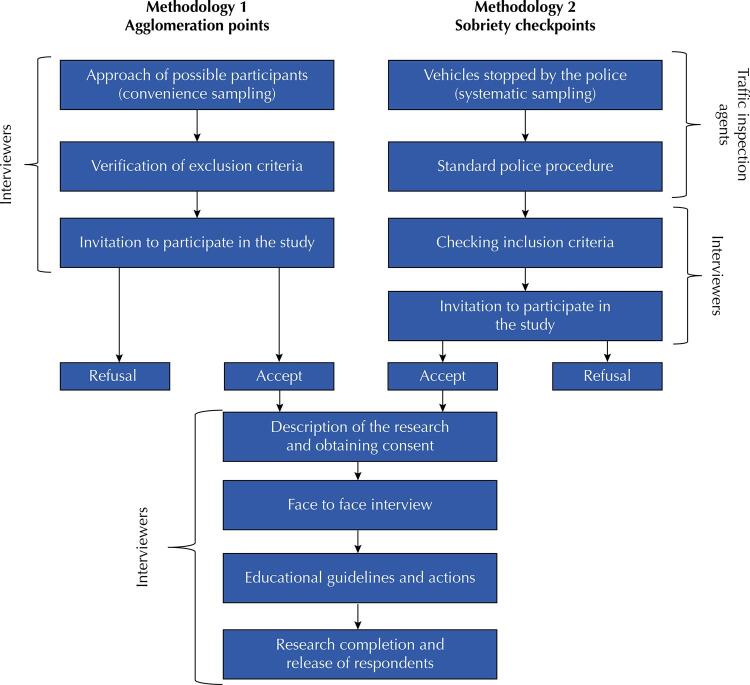
Research data collection flowchart - adapted from Pechansky et al. ^29^ (2012).

To obtain representative samples of drivers from each capital, two methodologies were used, with different samples ( Figure 1 ). Similar methodologies have already been implemented in countries such as Australia, Canada, the United States of America, among others ^27 , 28^ , using the same inclusion criteria but with different search questions. For both, the choice of approach locations was conducted in consensus with the agencies responsible for traffic inspection in each participating municipality. Support was provided by professionals from traffic safety agencies at municipal, state, and federal levels. Data collection was conducted from Wednesday to Sunday, at night, and, in some cities, also in the afternoon on Sunday.

### Selection of Participants

#### Methodology 1 - ‘agglomeration points’

For the first methodology, non-probabilistic convenience sampling was adopted. This was applied to ‘agglomeration points’ (AP) such as open markets, shopping malls, gas stations, and supermarkets. The selection of points was conducted with the consent of the owners, directors, and/or managers of each selected location. After a random approach by the interviewers, individuals who met the inclusion criteria were invited to participate in the study.

#### Methodology 2 - ‘sobriety checkpoints’

For the second methodology, data collection was performed at ‘sobriety checkpoints’ (SC), popularly known as ‘blitzes.’ SC installed on public roads with more intense traffic, with higher concentrations of bars, restaurants and nightclubs, and neighborhoods with different socioeconomic levels, were considered as recommended by Campos et al. ^28^ . In the selected locations, a systematic random sampling of vehicles/motorcycles was conducted, with one vehicle being selected in every five so that the driver was invited to participate in the study. After standard procedures performed by the police authority (verification of regularity and documentation of the vehicle and driver), drivers were invited to participate in the study ^27^ .

In both methodologies, individuals who met the inclusion criteria and agreed to participate signed an informed consent form. Guidelines were provided for them on the objectives, methods, benefits, and potential risks of participating in the research ^27^ . Then, data collection was performed individually by the interviewers, using a structured and standardized tool installed on tablet devices. After data collection, guidelines were provided on the risks of driving under the influence of alcohol ^27^ .

Those who agreed to participate signed an informed consent form. Then, the interview was conducted individually using a structured instrument. This instrument was developed by the authors of this study and comprises questions on sociodemographic characteristics, antecedents, and various risk factors for traffic accidents. The collected data were recorded on tablet devices during the interviews.

## Questionnaire and Indicator

The following questions from the questionnaire were used to compose the indicator that was used in this study:

In the last 30 days, did you consume at least one dose of alcoholic beverage? (Options: yes, no, don’t know/didn’t want to answer);

On any of those days when you consumed alcohol, did you drive after drinking? (Options: yes, no, don’t know/didn’t want to answer);

In the last 30 days, when you used alcohol and did not drive, what means of transport did you use to get around? (Options: Uber, other e-hailing apps, ride friend/family/partners, public transportation, taxi/mototaxi, other ways, don’t know/didn’t want to answer).

The indicator was calculated using the following formula: proportion of drivers who used Uber as a substitute for drinking and driving = (number of drivers who reported using Uber as a means of transport after drinking alcohol / number of drivers who reported drinking alcohol in the last 30 days and reported not driving after) * 100.

## Sample Size

Sample size calculations indicated that the number of respondents needed to compose the survey sample would be 296 individuals for each methodology (SC and AP) from each participating capital (n = 2,960), considering a significance level of 95% (α = 0.05), margin of error of 2.5 percentage points, prevalence of positive BAC of 4.2% ^29^ , and potential losses of 20%.

## Statistical Analysis of Data

Data were analyzed using Stata software program, version 16.0. The rake method was used to construct post-stratification weights using age and gender variables. To calculate the weights, the distribution of drivers by gender and age group, measured by the 2013 Brazilian National Health Survey, was used as a reference.

The proportion of drivers who used Uber as a substitute for drinking and driving, as well as the respective 95% confidence intervals (95%CI), were measured for total sample of each methodology and stratified by municipality, age group, gender, education level, and type of vehicle predominantly used by the interviewee (car or motorcycle). Fisher’s exact test was used to perform comparisons related to the magnitude of the indicator between different strata.

This research was approved by the Research Ethics Committee of the Universidade Federal de Goiás (no. 2,854,899/2018). The study was conducted in accordance with Resolution no. 466 of the Brazilian National Health Council, which regulates the standards for research on human beings in the country. This research was funded by the Ministry of Health of Brazil by a Decentralized Execution Term.

## RESULTS

In total, 8,864 car and motorcycle drivers from ten Brazilian capitals participating in the research were interviewed. Table 1 summarizes the number of interviewees approached for the survey according to the sample, as well as the rate of acceptance and completion of the interview. The overall acceptance rate was 93.6% and 97.7% among eligible drivers approached in AP and SC methodology, respectively.

**Table 1:** Number of eligible drivers approached, number of interviews conducted, and acceptance rate in the ten Brazilian capitals participating in the study.

Capital	Agglomeration points			Sobriety checkpoints		
	
	Number of eligible drivers approached	Number of interviews conducted	Percentage of acceptance	Number of eligible drivers approached	Number of interviews conducted	Percentage of acceptance
Belo Horizonte	335	302	90.1	387	380	98.2
Boa Vista	390	376	96.4	469	457	97.4
Cuiabá	420	387	92.1	415	408	98.3
Florianópolis	423	342	80.9	370	364	98.4
Macapá	595	575	96.6	589	581	98.6
Salvador	348	343	98.6	390	383	98.2
São Luís	398	392	98.5	521	513	98.5
São Paulo	430	426	99.1	452	440	97.3
Teresina	459	455	99.1	517	488	94.4
Vitória	533	455	85.4	423	414	97.9
**Total**	**4,331**	**4,053**	**93.6**	**4,533**	**4,428**	**97.7**

**Figure 2:**
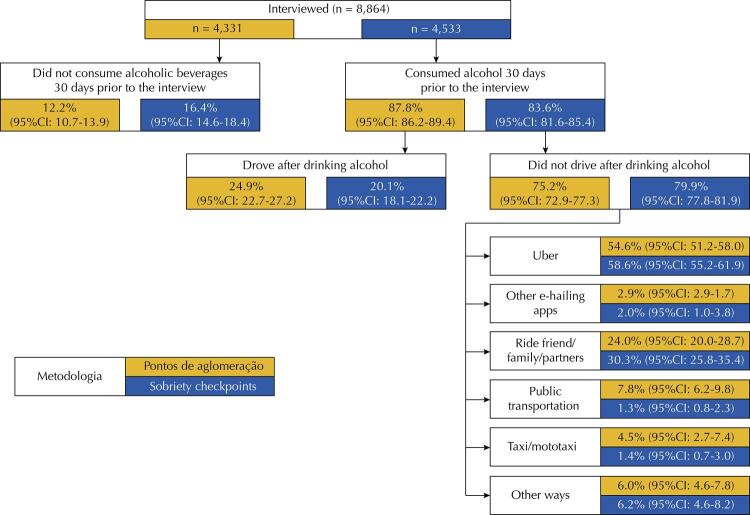
Distribution of respondents according to consumption of alcoholic beverages 30 days before the survey, driving after drinking, and means of transport used if they did not drive, according to the research methodology used, in the capitals participating in the study (2019).

Figure 2 shows the distribution of participants according to consumption of alcoholic beverages 30 days before the survey, driving after consumption and the means of transport used if they did not drive after drinking. Most drivers reported having consumed alcoholic beverages at least once in the 30 days before the survey (AP: 87.8%; 95%CI: 86.2–89.4. SC: 83.6%; 95%CI: 81.6–85.4). Most of these drivers, however, reported that they did not drive after drinking during the period (AP: 75.2%; 95%CI: 72.9–77.3. SC: 79.9%; 95%CI: 77.8–81.9). The most used means of transport to replace driving after drinking alcohol was the Uber system (AP: 54.6%; 95%CI: 51.2–58.0. SC: 58.6 %; 95%CI: 55.2–61.9).

95%CI: 95% confidence interval.

Note: did not want to answer: 0.3% (95%CI: 0.1%–1.3%) in agglomeration points and 0.3% (95%CI: 0.1%–0.7%) in sobriety checkpoints.

**Figure 3:**
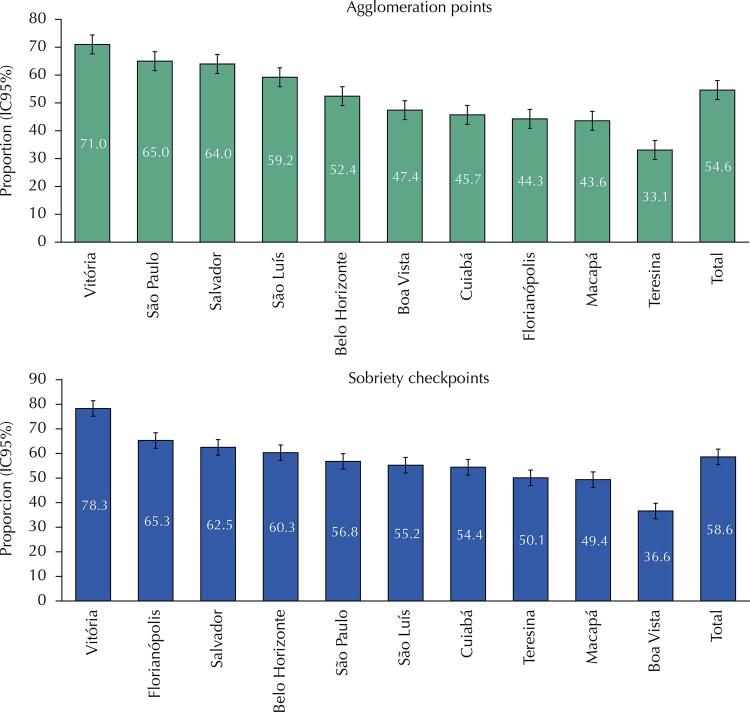
Proportion of drivers who used Uber after drinking alcohol according to the research methodology used in the capitals participating in the study (2019).

Figure 3 shows the proportion of drivers who used Uber after drinking alcohol, according to the methodology used and the municipality. According to the data collected using the AP methodology, the highest magnitude of this indicator was found in Vitória (71.0%; 95%CI: 63.5–77.5), whereas the lowest was observed in Teresina (33.1%; 95%CI: 22.7–45.5). According to the data collected using the SC methodology, the highest magnitude of the indicator was also found in Vitória (78.3%; 95%CI: 68.8–85.5), whereas the lowest magnitude was observed in Boa Vista (36.6%; 95%CI: 26.8–47.7).

Table 2 shows the proportion of drivers who used Uber after drinking, according to the methodology used and sociodemographic characteristics of drivers, for all participating capitals. It is possible to observe that the age group that presented the highest magnitude for this indicator was 18 to 29 years old (AP: 51.6%; 95%CI: 46.8–56.4. SC: 53.4%; 95%CI: 48.1–58.6). In addition, women (AP: 51.8%; 95%CI: 45.7–57.8. SC: 48.7%; 95%CI: 42.3–55.1) and the educational level corresponding to Higher education or more (AP: 51.8%; 95%CI: 46.5–57.2. SC: 46.6%; 95%CI: 42.4–50.8) were responsible for the highest magnitudes for this indicator. The AP methodology indicated greater magnitude of the indicator among motorcycle drivers (69.5%; 95%CI: 45.3–100.00), whereas the SC methodology indicated it among car drivers (44, 9%; 95%CI: 41.7–48.2).

**Table 2:** Proportion of drivers who used Uber after drinking alcohol according to the research methodology used and sociodemographic characteristics of drivers across all capitals participating in the study (2019).

Sociodemographic characteristics	Agglomeration points		Sobriety checkpoints	
	
	Proportion of drivers who used Uber after drinking alcohol (95%CI)	p-value	Proportion of drivers who used Uber after drinking alcohol (95%CI)	p-value
Age group (years)				
18–29	51.6 (46.8–56.4)	< 0.001	53.4 (48.1–58.6)	< 0.001
30–39	49.0 (43.0–55.0)	< 0.001	44.6 (39.6–49.7)	< 0.001
≥ 40	34.5 (26.7–39.7)	-	35.9 (31.2–40.9)	-
Gender				
Woman	51.8 (45.7–57.8)		48.7 (42.3–55.1)	
Man	40.6 (37.2–44.1)		41.1 (38.0–42.2)	
Education level				
Never studied/incomplete elementary school	25.1 (16.25–36.74)	< 0.001	16.8 (8.1–31.7)	< 0.001
Completed/incomplete elementary school	33.0 (24.6–42.6)	< 0.001	25.4 (16.3–37.2)	< 0.001
Completed high school/incomplete higher education	44.3 (40.0–48.6)	0.058	44.1 (39.5–48.8)	0.085
Higher education or more	51.8 (46.5–57.2)	-	46.6 (42.4–50.8)	-
Type of driver				
Car	61.4 (47.8–78.7)	0.048	44.9 (41.7–48.2)	0.028
Motorcycle	69.5 (45.3–100.0)	-	35.9 (28.0–44.7)	-

95%CI: 95% confidence interval.

## DISCUSSION

In this study, 83 to 87% of drivers reported drinking alcoholic beverages at least once in the 30 days before the survey. According to data from the 2013 Brazilian National Health Survey, the prevalence of consumption of alcoholic beverages once or more a week was 23.9% ^30^ . In 2019, the magnitude of this indicator increased to 26.4% ^31^ . Alcohol abuse, according to data from the same survey, increased from 13.7%, in 2013, to 17,1% in 2019 ^30 , 31^ . For all Brazilian capitals, according to data from Vigitel, the prevalence of alcohol abuse was even higher, reaching 18.8% in 2019, with a continuous increase in prior years ^32^ .

According to the data collected in this study, 20% to 25% of individuals who reported drinking alcohol stated that they did not drive after. This indicator, which measures the prevalence of driving after consumption of any amount of alcohol, is considered the most sensitive for monitoring this practice, as it is close to what is recommended in the *Lei Seca* ^6 , 9^ . This behavior was also identified by the 2019 National Health Survey, which estimated, among drivers over 18 years old, a prevalence of driving after consuming alcoholic beverages of 17.0% in Brazil and 14.0% in all capitals. Research conducted in European Union countries showed that 1 to 4% of drivers were driving under the influence of alcohol ^33^ . Although the comparison with global studies becomes difficult due to the different methodologies and legislation of the countries, higher prevalence is observed in Brazil when compared with Europe ^35^ .

Moreover, data from the Brazilian National Department of Transport Infrastructure show that, in 2017, 19,083 drivers were caught by the Federal Highway Police driving under the influence of alcohol. During this period, about 6,450 accidents caused by drunk drivers were recorded on federal highways, with more than 13,000 victims and about 1,000 deaths ^36^ . Statistics show that driving a vehicle after drinking alcohol remains a serious problem in the country.

Data from the survey showed that the Uber system was the most used means of transport to replace driving after drinking alcohol, ranging from 55% to 59%. Factors such as low cost, convenience, simple payment method, accessibility, and security may explain this preference ^13 , 18 , 23 , 37^ . According to Martin-Buck ^18^ , e-hailing services have led to a 10% to 11.4% reduction in alcohol-related fatal car accidents. In New York and in 15 Illinois counties, a reduction in the number of people who drove under the influence of alcohol was observed after Uber system implementation ^19^ . A study conducted in Toronto (Canada) found that the main reason for using e-haling services was to travel to entertainment places, bars, and other activities related to alcoholic beverages consumption, possibly reducing the prevalence of driving after drinking alcohol ^37^ .

In a study conducted in four cities in the United States, a reduction of up to 61.8% was observed in the rate of RTI related to alcohol use (an absolute decrease of 3.1 accidents per week) after the resumption of Uber system activities in these cities ^21^ . Dills and Mulholland observed a 17% to 40% reduction in RTI mortality in US counties after four years or more since implementing the Uber system ^22^ . In fact, based on the results of these and other studies, several agencies responsible for traffic safety emphasize the need for public policies aimed at improving the availability, convenience, and accessibility of this type of passenger transport service ^38 , 39^ .

On the other hand, the study by Brazil and Kirk did not note any impact of the Uber system on RTI morbidity and mortality in the United States ^17^ . Barrios et al. found that, in the United States, the use of e-hailing services was associated with an increase of approximately 3% in the number of deaths and fatal accidents involving motor vehicles. This increase occurred not only for vehicle occupants, but also for pedestrians ^24^ . A study conducted by Brazil and Kirk showed that the availability of Uber system is not associated with changes in total traffic fatalities nor with a reduction in driving under the influence of alcohol. On the contrary, it showed an association with the increase in traffic fatalities in urban and highly populated municipalities ^40^ .

In Brazilian capitals, the study showed a higher proportion of drivers who did not drive and used the Uber app to travel after drinking alcohol. In Vitória, the prevalence ranged from 71% to 78%. To Tirachini and Río ^41^ , the frequency of use of e-hailing applications is not associated with their implementation time and availability of cars. According to data from the Brazilian National Health Survey, Vitória was the Brazilian capital with the lowest prevalence of driving after drinking alcohol in 2013 (9.9%) ^30^ and the fourth in 2019 (9.2%) ^31^ , with a downward trend.

The lowest proportion of drivers who used Uber after drinking alcohol was observed in Teresina (33%) and Boa Vista (37%). Data from the Brazilian National Health Survey show that these were the capitals with the highest prevalence of driving after drinking alcohol in 2013 (44.8% in Teresina and 35.6% in Boa Vista) ^30^ . In 2019, these capitals had the fourth and fifth highest magnitudes for this indicator (28.5% in Teresina and 28.5% in Boa Vista) ^31^ . Considering the years 2000 to 2016, among Brazilian capitals, the highest mortality rate from land transport accidents occurred in Boa Vista (41.6 deaths/100,000 inhabitants). As for occupants of motorcycles or tricycles, the highest mortality also occurred in Boa Vista (16.1/100,000 inhabitants) ^42^ .

The age group that showed the highest proportion of drivers who used Uber after drinking alcohol was 18 to 29 years old (52% to 53%). These results differ from those found in studies conducted in developed countries, which showed a higher prevalence of driving after drinking alcohol precisely among young adults ^43 , 44^ . However, investigations conducted in Brazil showed results consistent with those of this study, indicating that 30 years or older drivers are more likely to drive under the influence of alcohol ^27 , 28 , 45^ . A study by Guimarães and Morais Neto ^45^ indicated a higher prevalence of driving under the influence of alcohol in the 30 to 39 age group throughout Brazil and in Southeast and South macro-regions. This behavior may reflect greater car availability suitable for this age group and lower adherence of this population to traffic safety measures since their bad habits are more consolidated ^45^ .

In addition, women were responsible for the highest proportion of drivers who used Uber after drinking alcohol (AP: 51.8%; 95%CI: 45.7–57.8. SC: 48.7%; 95%CI: 42.3–55.1). These results are consistent with those observed in several studies, which report that men tend to engage in risky health behaviors more often than women and are the main victims of traffic accidents ^2 , 4 , 46^ . A study conducted by Andrade et al. ^46^ among medical students in the southern region of Brazil revealed that young men reported a higher frequency of risk behaviors such as learning to drive a car when they were 16 years old or less and having consumed alcohol before driving a vehicle 30 days prior to the survey. According to data from the 2019 Brazilian National Health Survey, 20.5% of men reported that they had driven a car or motorcycle after drinking alcohol in the 12 months before the survey. On the other hand, only 7.8% of women revealed this type of behavior ^31^ . The risk levels of mortality from RTI are much lower for women compared to men ^47^ .

The education level higher education or more was the one with the highest proportion of drivers who used Uber after drinking alcohol (47% to 52%). The prevalence of abusive alcoholic beverages consumption is inversely related to the education level (more years of study leads to lower prevalence of abusive consumption) ^31^ . Paradoxically, data from the 2019 PNS also showed that the prevalence of alcohol consumption once or more times a week is directly proportional to the education level (more years of study leads to greater prevalence of consumption) ^31^ . The same occurs with the indicator analyzed here: the proportion of drivers who did not drive and used Uber after drinking alcohol increased with education level. Although it may seem as a contradiction, these findings can be explained by the fact that there is an increased risk of alcohol abuse and/or dependence among populations of lower socioeconomic status ^48 , 49^ . Thus, although alcohol consumption is less frequent among populations of lower socioeconomic status, they are more likely to be negatively affected by alcohol if they drink, which can lead to abusive consumption, risky behavior in traffic, dependence, and death ^50^ .

The AP methodology indicated greater magnitude of the indicator among motorcycle drivers (AP = 69.5%; 95%CI: 45.3–100.00), whereas the SC methodology indicated it among car drivers (SC = 44.9%; 95%CI: 41.7–48.2). This observation may have occurred since the number of motorcycle drivers participating in the SC methodology was very small, representing only 23.6% of respondents. Meanwhile, in the AP methodology, this type of conductor represented 36.4% of the total number of respondents. The fact that commercial vehicle drivers were not included in the survey may have impacted this fact since most motorcycle drivers approached in the SC methodology fitted this profile.

Knowledge about the proportion of Uber use instead of driving after drinking alcohol may contribute to actions to prevent morbidity and mortality in traffic. This type of scientific evidence on factors associated with RTI presents the potential to guide interventions in Brazil toward reaching the goal of a 50% reduction in traffic deaths and injuries by 2030, as defined in the Brazilian National Plan for the Reduction of Deaths and Injuries in Traffic (PNATRANS), part of the Sustainable Development Goals.
